# Local hyperactivation of L-type Ca^2+^ channels increases spontaneous Ca^2+^ release activity and cellular hypertrophy in right ventricular myocytes from heart failure rats

**DOI:** 10.1038/s41598-021-84275-w

**Published:** 2021-03-01

**Authors:** Roman Y. Medvedev, Jose L. Sanchez-Alonso, Catherine A. Mansfield, Aleksandra Judina, Alice J. Francis, Christina Pagiatakis, Natalia Trayanova, Alexey V. Glukhov, Michele Miragoli, Giuseppe Faggian, Julia Gorelik

**Affiliations:** 1 National Heart and Lung Institute, Imperial College London, Du Cane Road, London, W12 0NN UK; 2 Dipartimento Di Cardiochirurgia, Università Degli Studi Di Verona, Ospedale Borgo Trento, P.le Stefani 1, 37126 Verona, Italy; 3 Department of Medicine, Cardiovascular Medicine, Madison School of Medicine and Public Health, University of Wisconsin, Madison, WI 53705 USA; 4 Humanitas Clinical and Research Center - IRCCS, Rozzano, MI Italy; 5 Department of Biomedical Engineering and Alliance for Cardiovascular Diagnostic and Treatment Innovation, Johns Hopkins University, Baltimore, USA; 6 Dipartimento Di Medicina E Chirurgia, Università Degli Studi di Parma, Via Gramsci 14, 43124 Parma, Italy

**Keywords:** Cardiovascular models, Cardiovascular biology, Mechanisms of disease, Calcium signalling, Single-channel recording

## Abstract

Right ventricle (RV) dysfunction is an independent predictor of patient survival in heart failure (HF). However, the mechanisms of RV progression towards failing are not well understood. We studied cellular mechanisms of RV remodelling in a rat model of left ventricle myocardial infarction (MI)-caused HF. RV myocytes from HF rats show significant cellular hypertrophy accompanied with a disruption of transverse-axial tubular network and surface flattening. Functionally these cells exhibit higher contractility with lower Ca^2+^ transients. The structural changes in HF RV myocytes correlate with more frequent spontaneous Ca^2+^ release activity than in control RV myocytes. This is accompanied by hyperactivated L-type Ca^2+^ channels (LTCCs) located specifically in the T-tubules of HF RV myocytes. The increased open probability of tubular LTCCs and Ca^2+^ sparks activation is linked to protein kinase A-mediated channel phosphorylation that occurs locally in T-tubules. Thus, our approach revealed that alterations in RV myocytes in heart failure are specifically localized in microdomains. Our findings may indicate the development of compensatory, though potentially arrhythmogenic, RV remodelling in the setting of LV failure. These data will foster better understanding of mechanisms of heart failure and it could promote an optimized treatment of patients.

## Introduction

Heart failure (HF) caused by myocardial infarction (MI) is a major cause of hospitalization and mortality worldwide^1^. The increased arrhythmogenic propensity after MI is responsible for more than 50% of deaths among patients with HF^2^. In recent years, right ventricle (RV) dysfunction (reduced ejection fraction) has been shown to be relatively frequent in patients with HF^3,4^. RV dysfunction in HF is thought to arise from several factors, including ischemia/infarct of the RV, septal dyssynergia, pulmonary hypertension, neurohormonal activation, and inflammation^5,6^. Moreover, patients with left ventricle (LV) systolic dysfunction and HF who develop RV failure have worse prognosis and their average survival is usually less than two years^7–9^. Thus, elucidating why the RV function is declined in the settings of LV disorder has a particular importance in understanding the mechanisms of HF.

Pathophysiological remodelling in HF occurs at multiple levels. Recent studies of LV dysfunction in HF showed that the progressive disorganization of Ca^2+^-signalling microdomains disrupts excitation–contraction coupling, suppressing cardiomyocyte contractility^10^, and promotes the development of arrhythmogenic triggers at the whole heart level^11^. Ventricular myocytes have a well-organized transverse-axial tubular (TAT) network, which brings L-type Ca^2+^ channels (LTCCs) in close proximity to clusters of ryanodine receptors 2 (RyR2) on the sarcoplasmic reticulum, allowing for a synchronous contraction of the cell. Disruption of TAT network and flattening of sarcolemma membrane in HF can provoke a deranged intracellular Ca^2+^ handling and malignant arrhythmias^12,13^. Specifically, in failing LV myocytes (LVMs) LTCC relocate from their predominant localization in T-tubules to the surface membrane (crest) where they can become hyperphosphorylated^11^. TAT network loss in failing LVMs promotes “orphaning” of RyR2 and facilitates the dyssynchronization of sarcoplasmic reticulum Ca^2+^ release^14–16^.

In this work, we used a combination of structural and functional studies to probe the microdomain-specific alteration in local Ca^2+^ handling in failing RVMs. We showed that 16 weeks after the left ventricle coronary arterial ligation caused HF, RVMs become hypertrophied with a higher contractility, and showed significant structural changes including a less regular TAT network and a flatter cell surface topography. While the distribution of functional LTCCs in RVMs was preserved in HF, the open probability (Po) of LTCCs was dramatically increased in T-tubules of failing myocytes. The localized increase in LTCC Po was accompanied by an enhanced frequency and a size of spontaneous Ca^2+^ sparks observed in failing RVMs resulting in elevated local Ca^2+^ wave frequency. Our findings may indicate the development of compensatory, though potentially arrhythmogenic, RV remodelling in the setting of LV failure. This work provides new insights into nanoscale level of RVMs remodelling in HF.

## Results

### Reduction of the TAT network organization in RVMs from MI rats

**Figure 1:**
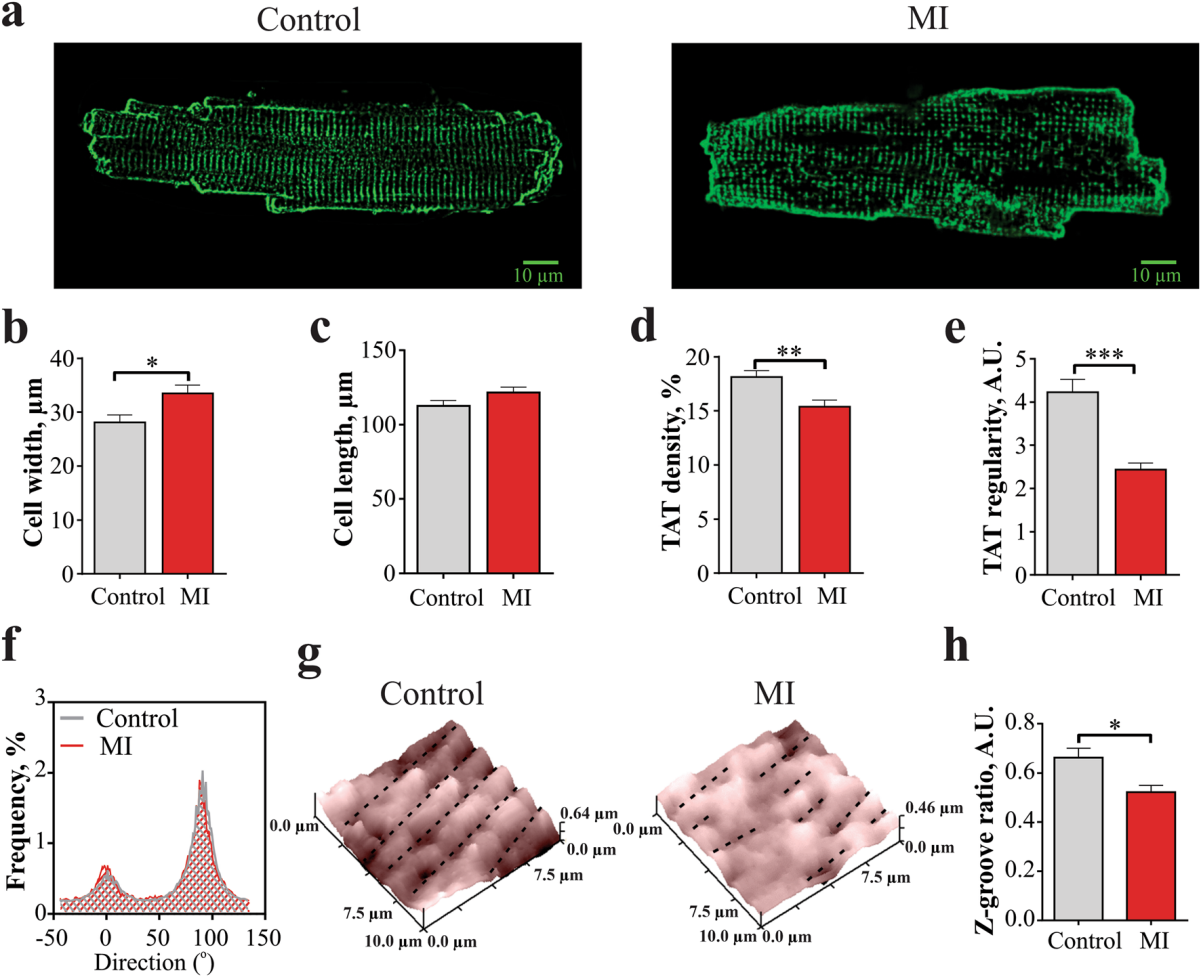
Structural changes of RVM membrane organization 16 weeks after MI. **(a)** Example images of control (left) and MI (right) RVM. **(b)** Average width and **(c)** length of RVM isolated from control and MI rats. Control n = 53 cells from 5 rats, MI n = 63 from 6 rats. *P < 0.05, by unpaired Student t test. (**d)** TAT network average density and **(e)** TAT power of regularity measured in control and MI RVMs. Control n = 40 cells from 5 rats, MI n = 62 from 6 rats. **P < 0.01, ***P < 0.001, by unpaired Student t test. **(f)** Directionality histograms for the TAT network in control and MI RVMs. **(g)** Representative 10 × 10 µm surface scans of control and MI RVMs obtained by SICM. **(h)** Average z-groove ratio measured in control and MI RVMs. Control n = 26 cells from 6 rats, MI n = 37 from 5 rats. *P < 0.05 by unpaired Student t test.

We used confocal microscopy on freshly isolated myocytes stained with Di-8-ANEPPS to visualize the internal TAT structure. Representative TAT images from control and MI RVMs are shown in Fig. 1a. We found that MI RVMs were 18% wider than control cells (p < 0.001, Fig. 1b), and only slightly longer (Fig. 1c) confirming the progression to hypertrophy. Analysis of the TAT network demonstrated (Supplementary Fig. S1) that MI RVMs had an 11% lower density as compared to control RVMs (p < 0.001, Fig. 1d). The regularity of the TAT network in MI RVMs was significantly (by 42%, p < 0.001) decreased compared with control RVMs (Fig. 1e). Recent studies by Schobesberger et al. showed a progressive increase of axial tubules in LVM during transition from healthy to hypertrophic state during HF development in a similar MI rat model^17^. However, using directional analysis of T-tubule alignment^18^, we did not find any changes in T-tubule fraction oriented transversally and axially in control versus MI RVMs (Fig. 1f and Supplementary Fig. S2).

The surface topography of RVMs was also altered after 16 weeks post-MI as it was reported previously for HF LV cardiomyocytes^11,17^. Representative 10 × 10 µm surface scans of control and MI RVMs are shown in Fig. 1g. We used Z-groove ratio as a measure of surface regularity, as previously described^19^. RVMs from MI rats showed a significant reduction in the Z-groove ratio of 27%, when compared to control cells (p < 0.001, Fig. 1h).

### MI RVMs exhibit larger contractions with smaller Ca^2+^ transients

**Figure 2:**
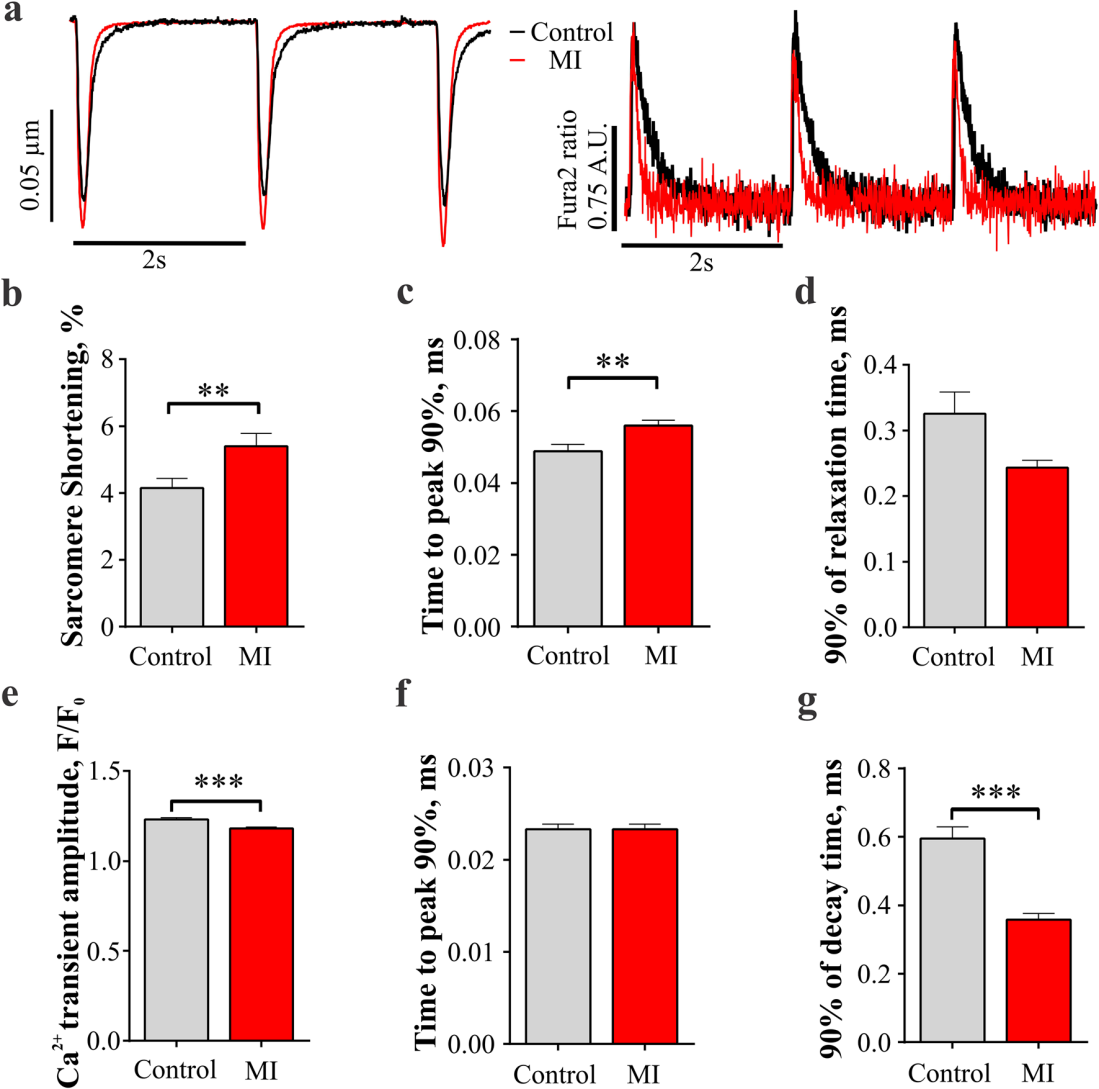
Sarcomere shortening and stimulated Ca^2+^ transients in MI RVMs. **(a)** Representative rat sarcomere shortenings recordings and Ca^2+^ transients traces in control and MI RVMs. **(a)** Average sarcomere shortening amplitude , **(b)** time to 90% peak (TTP90) and **(c)** time from peak to 10% of baseline (TTB90) measured at 0.5 Hz for both control and MI RVMs. Control n = 104 cells from 4 rats, MI n = 74 from 3 rats. **P < 0.01, by Mann–Whitney test. **(e)** Amplitude of calcium transient (F/F_0_), **(f)** time to 90% peak from 10% baseline (TTP90) and **(g)** time from peak to 10% of baseline (90% decay time) were measured at 0.5 Hz in control and MI RVMs. Control n = 158 cells from 4 rats, MI n = 95 from 3 rats. ***P < 0.001, by Mann–Whitney test.

To address functional changes in RVM, we applied CytoCypher high throughput system to measure cellular contractility and Ca^2+^ transients with the fluorescent probe Fura-2AM. Figure 2a shows representative Ca^2+^ transients and cell shortening recordings evoked in control and MI RVMs at 0.5 Hz field stimulation. MI RVMs showed significantly higher sarcomere shortening amplitude (p < 0.01, Fig. 2b) and longer rise time (p < 0.01, Fig. 2c) as compared to control RVMs, without a significant change in the relaxation time (Fig. 2d).

The amplitude of Ca^2+^ transients was reduced in MI RVMs (p < 0.01, Fig. 2e). There were no differences in Ca^2+^ transient rise time between control and MI RVMs (Fig. 2f). Decay time was significantly shorter in MI RVMs (p < 0.001, Fig. 2g).

### Spontaneous Ca^2+^ activity is increased in RVMs after MI

**Figure 3:**
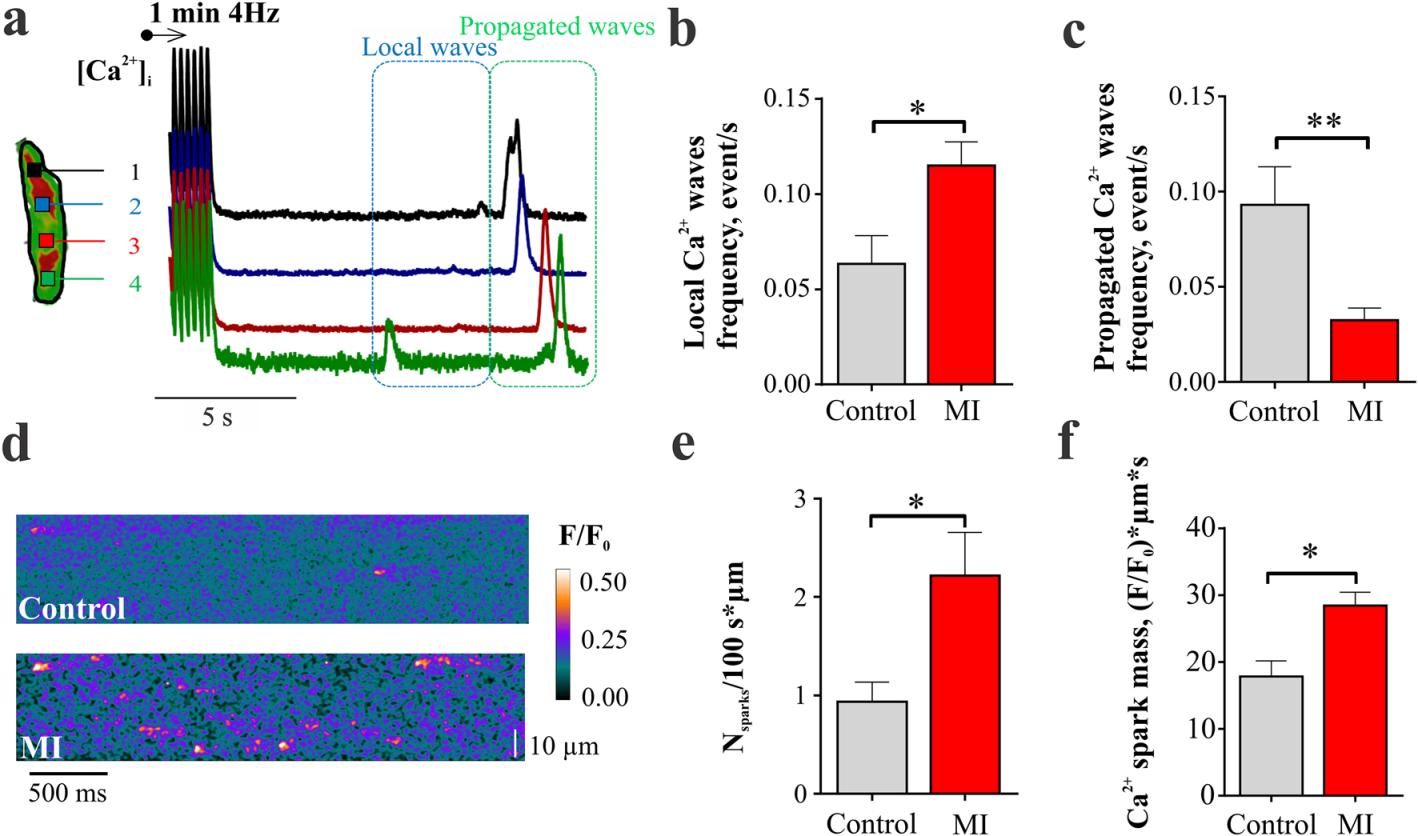
Spontaneous Ca^2+^ activity in MI versus control RVMs. **(a)** Illustration of the spontaneous Ca^2+^ waves recorded in control and MI RVMs. Myocytes were paced for 1 min at 4 Hz to enhance SR Ca^2+^ loading, then the spontaneous Ca^2+^ activity was recorded during 16 s after the pacing cessation. Propagated and local Ca^2+^ waves were analysed separately. **(b)** Analysis of local and **(c)** propagated Ca^2+^ wave frequency in control and MI RVM. Control n = 10 cells from 4 rats, MI n = 13 cells from 4 rats. **P < 0.01, by unpaired Student t test. **(d)** Line-scan images of Ca^2+^ sparks activity in RVMs from control and MI rats. **(e)** Analysis of average Ca^2+^ sparks frequency and **(f)** mass (amplitude x full width at half maximum x full duration at half maximum) in control and MI RVMs. Control n = 26 cells from 4 rats, MI n = 21 from 4 rats. *P < 0.05, by Mann–Whitney test (frequency) and unpaired Student t test (mass).

Spontaneous Ca^2+^ waves were recorded in single RVMs after 1 min of 4 Hz pacing. Representative optical [Ca^2+^]_*i*_ traces recorded at 4 consecutive locations in a selected myocyte are presented in Fig. 3a. Two types of spontaneous Ca^2+^ release events were distinguished: local (i.e., non-propagating) and propagated Ca^2+^ waves. The frequency of local Ca^2+^ waves attributed to a local cluster of Ca^2+^ sparks was 80% higher in MI than in control RVM (p < 0.05, Fig. 3b) while the frequency of propagated Ca^2+^ was decreased in MI versus healthy RVMs (Fig. 3c). Local spontaneous Ca^2+^ activity was further analysed by a high-resolution line-scan imaging of single Ca^2+^ sparks. Examples of Ca^2+^ spark activity in a control and an MI RVM are presented in Fig. 3d. The frequency of Ca^2+^ sparks was almost 2 times higher in MI RVMs as compared to control cells (p < 0.05, Fig. 3e). Moreover, the mass of Ca^2+^ sparks, calculated as amplitude multiplied by half maximum width multiplied by half maximum duration^20^, was significantly higher in MI RVMs (p < 0.05, Fig. 3f) supporting a significant increase in local Ca^2+^ waves (Fig. 3b) and indicating a local disruption of Ca^2+^ handling. The higher Ca^2+^ sparks mass in MI myocytes was a result of significant increase of Ca^2+^ spark amplitude and width (Supplementary Fig. S3).

### Abnormal localization and function of L-type Ca^2+^ channels in RVM after MI

**Figure 4:**
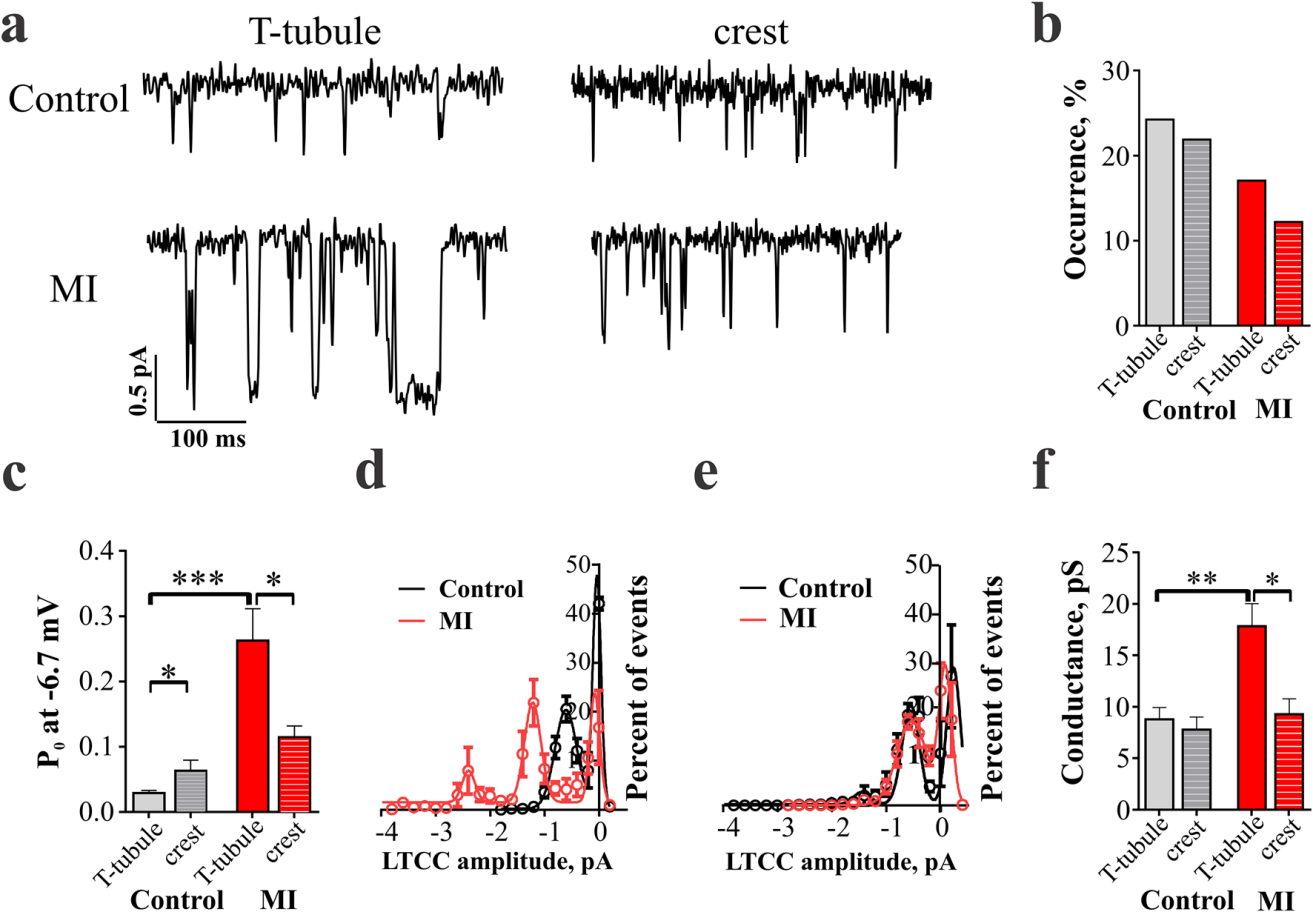
L-type Ca^2+^ channel properties in MI RVMs. **(a)** Representative traces of LTCCs recorded from a holding potential of -96.7 to a depolarizing step of -6.7 mV in T-tubule or crest from control and MI RVMs. **(b)** LTCC percentage of occurrence at T-tubule and crest regions from control and MI RVMs. Control T-tubule n = 66 cells, crest n = 64 cells; MI T-tubule n = 41 cells, crest n = 41 cells. **(c)** Open probability (Po) of LTCC at a voltage step of -6.7 mV from control and MI RVMs. Control T-tubule n = 7 cells, crest n = 8 cells; MI T-tubule n = 7 cells, crest n = 5 cells. *P < 0.05, ***P < 0.001, by Kruskal–Wallis test. **(d)** Amplitude histograms of LTCC (dots) recorded at depolarizing step of -6.7 mV and fit with multicomponent Gaussian functions (connecting curves). Histograms were constructed from n = 6–10 channel activity. LTCC activity was analysed separately for T-tubule and **(e)** crest channels. **(f)** Conductance of LTCC located in T-tubule or crest regions of RVMs. Control T-tubule n = 7, crest n = 10; MI T-tubule n = 10, crest n = 12. *P < 0.05, **P < 0.01, by Kruskal–Wallis test.

To characterize changes in local Ca^2+^ signalling, we applied super-resolution scanning patch clamp technique to measure microdomain-specific LTCC activity as described previously^11^. Scanning patch-clamp was used to assess the distribution and biophysical properties of single LTCC both in T-tubules and on the crest sarcolemma. Representative traces from LTCC recordings are shown in Fig. 4a. In control RVMs, the chance to obtain LTCC current in the patch (i.e., channel occurrence) was similar between T-tubule and crest regions (Fig. 4b). In MI RVMs, however, the LTCC occurrence was decreased in both regions: LTCC had a 30% and 44% lower occurrence in the T-tubule and crest, respectively, when compared to the same regions in control RVMs.

The open probability (Po) of LTCC at T-tubule and crest was analysed at the activation step of -6.7 mV. In control RVMs, the Po of LTCC from T-tubules was significantly lower as compared to the Po of LTCC from crest (p < 0.05, Fig. 4c). Interestingly, in MI RVMs, LTCC located at the T-tubules had a significantly enhanced Po as compared to both the control T-tubule (p < 0.001 vs. control) and the MI crest channels (p < 0.05 vs. MI T-tubule). All-points histograms revealed that single channel amplitude was elevated in T-tubule from 0.59 ± 0.17pA in control to 1.22 ± 0.17pA in MI RVMs and multi-channel openings occurred more likely in T-tubule of MI than control myocytes (Fig. 4d) suggesting an increase in the channel interactions in this microdomain on MI cells. Representative traces and current–voltage relationships for the LTCC located in T-tubule and crest of RVMs are presented in (Supplementary Figs. S4 and S5). From these plots, the average single-channel conductance was calculated, showing that it was also significantly elevated in MI T-tubule LTCC as compared to control T-tubule LTCC (p < 0.01, Fig. 4f). In contrast, no changes in single channel Po or amplitude were found for crest LTCCs (Fig. 4e,f) highlighting the T-tubule localized remodelling of Ca^2+^ signalling.

### Protein kinase A-dependent phosphorylation of LTCC increases activity of T-tubule channels in MI RVM

**Figure 5:**
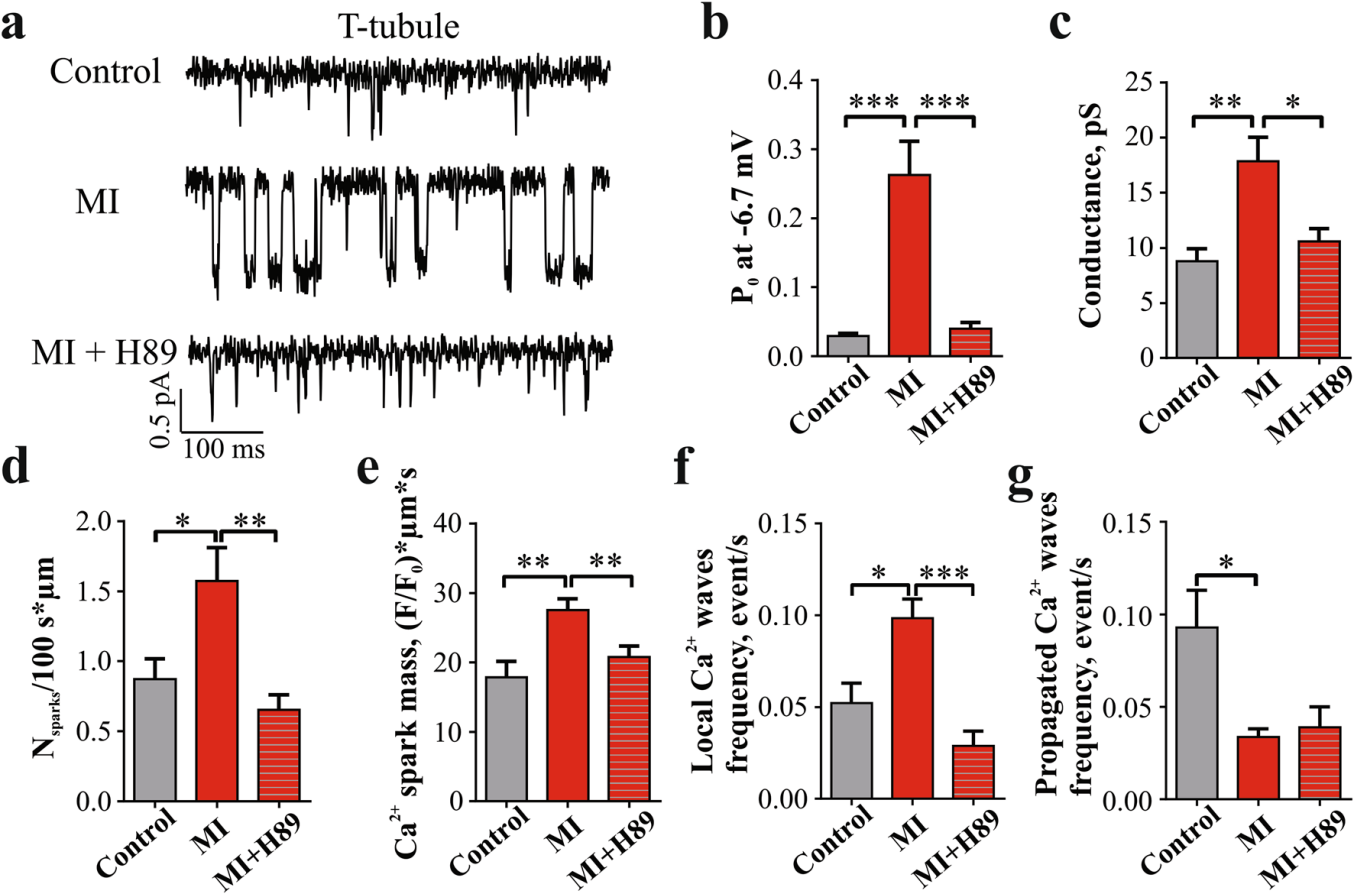
PKA blocker H89 reduced the Po of LTCCs from the T-tubules and reduced spontaneous Ca^2+^ spark rate in MI RVMs. **(a)** Representative traces of single LTCC activity in control, MI, and MI treated with H89. **(b)** Statistical analysis of open probability of LTCCs located in T-tubules of control, MI, and MI treated with H89. Control T-tubule n = 7 cells, MI T-tubule n = 7 cells, MI + H89 T-tubule n = 5 cells. ***P < 0.001, by Kruskal–Wallis test. **(c)** Conductance of single LTCCs located in T-tubules of control, MI, and MI treated with H89 RVMs. Control T-tubule n = 7 cells, MI T-tubule n = 7 cells, MI + H89 T-tubule n = 4 cells. *P < 0.05, **P < 0.01 by Kruskal–Wallis test. **(d)** Histograms shoving the effect of PKA blockage by H89 on Ca^2+^ sparks frequency and **(e)** Ca^2+^ sparks mass**.** Control n = 26 cells from 4 rats, MI n = 44 from 7 rats, MI + H89 n = 25 from 3 rats. *P < 0.05, by one-way ANOVA test. **(f)** Analysis of H89 effect on the frequency of local and **(g)** propagated Ca^2+^ waves in MI RVM. Control n = 10 cells from 4 rats, MI n = 24 cells from 4 rats, MI + H89 n = 10 from 3 rats, *P < 0.05, ***P < 0.001, by one-way ANOVA test.

Increase in LTCC Po has been linked to channel phosphorylation, and PKA was shown to be one of the main phosphorylation agents of Ca^2+^ handling proteins, including LTCC^21,22^. A recent study of human LVMs from ischemic cardiomyopathy hearts showed that the hyperactive T-tubule anchored LTCCs can be phosphorylated by PKA^23^. To assess if PKA is involved in the elevated Po observed in T-tubule LTCC in MI RVM, we used H89 as a PKA blocker. Traces of LTCC activity recorded at − 6.7 mV in control, untreated MI, and H89-treated MI RVMs are presented in Fig. 5a. Incubation of MI RVMs with 10 µM H89 significantly reduced the Po of LTCC located at T-tubules (p < 0.001 vs MI T-tubule LTCCs) and restored the control values (Fig. 5b). The average conductance of LTCC located in T-tubules of MI RVMs treated with H89 was also significantly decreased to conductance values similar to those of LTCC in control T-tubule (p < 0.05 vs MI T-tubule, Fig. 5c). The cooperative LTCC activation observed in MI TT LTCCs was also reduced to control values after the PKA blockage (Supplementary Fig. S6).

To elucidate the impact of PKA phosphorylation on spontaneous Ca^2+^ spark and Ca^2+^ wave activities we measured them in the presence of H89. Application of H89 returned the frequency of Ca^2+^ sparks recorded in line-scan to control levels (Fig. 5d) and reduced Ca^2+^ spark mass (Fig. 5e). The enhancement of local Ca^2+^ waves observed in MI RVMs has also returned to control levels after H89 treatment (Fig. 5f). However, blocking PKA did not change the frequency of propagated Ca^2+^ waves (Fig. 5g).

## Discussion

This study presents microdomain-specific changes in RVMs after LV MI, highlighting cardiomyocyte microarchitecture remodelling and associated localized disturbances of Ca^2+^ handling. The major finding of this study is that disruption in the membrane organization after MI leads to PKA-dependent hyperactivation of a sub-population of LTCCs located in T-tubules in RVMs which is accompanied by elevated activity of spontaneous Ca^2+^ release from the sarcoplasmic reticulum Ca^2+^ stores and altogether can contribute to hypertrophic remodelling of the RV contractility.

Here we provide the first evidence of a hypertrophic remodelling in RVMs from a LV MI model. It has been shown in LVMs^11^ that a similar structural remodelling is associated with a rearrangement of LTCC location with a concomitant increase in Po in the crest microdomain. In contrast, the results of the present study show, in the same animal model, that in RVMs, the T-tubule microdomain is the one containing hyperactive LTCCs that show an increased Po linked to enhanced PKA activity.

We found a significant hypertrophic remodelling of RV myocytes including cell widening, reduction of TAT network density and regularity after MI (Fig. 1d,e). Degradation of the TAT network is frequently observed under stress and elevated workload^24^, potentially precipitated by cell enlargement and increased neurohormonal activation^6,25^. Stefanon et al.^26^ also observed increased collagen deposition in the extracellular matrix in the RV after MI, which leads to fibrosis and could potentially drive dilatation and loss of T-tubules^27^. Investigation of TAT reorganization in LVMs during progression of HF^17^ showed an initial increase in the number of axial elements, however it reverted back at the late stages of the disease. In the current study, alignment analysis of TAT did not show such change, as similar proportions of axial and transverse tubules in RVMs after MI were found (Fig. 1f). Our study revealed that degradation of TAT network in RVMs was accompanied with the flattening of the sarcolemma z-grooves, similar to observations in LVMs^10,11^. These findings suggest a general mechanism underlying the impaired function of failing myocytes, which is related to the loss of local organization and signalling.

Functionally, MI RVMs show larger sarcomere shortening and longer time to peak (Fig. 2b,c). Ca^2+^ transients exhibit slightly lower amplitude and shorter decay time (Fig. 2e,g). Observed modulation of contraction and Ca^2+^ transients in MI RVMs could be due to the hypertrophic remodelling happening in the cells^28^, together with the response to the high sympathetic stimulation present in heart failure^29^. Increased contractility could be promoted by the microtubule proliferation observed in hypertrophic RVMs^30^. Enhanced sympathetic stimulation in MI rat could be responsible for the inotropic effects on contraction and Ca^2+^ transients^31^. Structural remodelling can produce a disconnection of LTCC and RyR2 with a desynchronization of Ca^2+^ transients and prolongation of contraction^32^.

We showed that LTCCs localized in T-tubule and crest regions of failing RVMs are subjected to different control mechanisms. LTCCs in T-tubules, which take part in the coupling with RyR2 and participate in Ca^2+^-induced Ca^2+^ release show a significantly elevated Po and single channel conductance in MI (Fig. 4c–f). Such differences in the local control of LTCC activity have been previously reported for the failing LVMs, mostly for the crest membrane fraction of LTCC^11,33^, but also for the T-tubule fraction in ischemic cardiomyopathy^23^. In myocytes, AKAP5 was shown to organise the signalling pathway following sympathetic stimulation by targeting adenylyl cyclase, PKA and calcineurin to a specific subpopulation of LTCC^34^. In mice with AKAP5 knockout, sympathetic stimulation induced phosphorylation of all subpopulations of LTCCs by the activity of freely diffusing PKA^34^. This could suggest that only the T-tubular LTCCs are associated with AKAP5 and thus activated by PKA phosphorylation.

We observed an increased cooperative activation of LTCC. More channels were recorded in MI patches, with mean amplitude in the T-tubule channels of MI almost twice as big as in control (Fig. 4d). Moreover these LTCC alterations in MI were brought back to control levels after PKA blockade (Fig. 5 and Supplementary Fig. S6). Similar behaviour LTCC was observed by Ito et al.^35^ following β-adrenergic stimulation including an occurrence of higher numbers of functional channels on the membrane and a cooperative activation of LTCC. The authors propose that channels redistribute in the membrane and multichannel functional complexes form via C-terminal-to-C-terminal interactions^36^. This mechanism could be related to observed changes in LTCC behaviour in MI T-tubules.

Such augmentation of PKA activity in T-tubules may also contribute to a higher phosphorylation of RyR2^34^ increasing their sensitivity cytosolic and/or luminal [Ca^2+^] and further facilitating SR Ca^2+^ leak^37^. Our data confirmed that blockade of PKA in MI RVMs returns the frequency of Ca^2+^ sparks and Ca^2+^ waves to the control levels (Fig. 5d,e). Increased frequency of Ca^2+^ sparks and local Ca^2+^ waves along with a higher average Ca^2+^ spark mass could induce local rises of [Ca^2+^] triggering Na^+^/Ca^2+^ exchanger and, consequently, arrhythmogenic delayed afterdepolarization activity in MI RVMs^38^. The reduction of propagated Ca^2+^ wave frequency observed in MI RVMs could be due to the elevated rate of Ca^2+^ sequestration in SR via PKA increased phosphorylation of phospholamban and SERCA2 activation^39^. Whether the arrhythmogenesis of RVMs in MI is compensated by the reduction in the propagated Ca^2+^ wave frequency needs to be addressed in further studies.

In conclusion, we found a hypertrophic remodelling of RVMs in MI that is associated with enhanced PKA activation and membrane remodelling. Both of these factors could be a result of the adaptive remodelling of RV in the settings of chronic MI and can contribute to the altered contractility and Ca^2+^ cycling. Observed local regulation of LTCC and RyR2 by PKA could potentially help in understanding the mechanisms of RV adaptation to the disease.

## Methods

### Ethics and myocyte isolation

All animal experiments were carried out in accordance with the United Kingdom Home Office Animals (Scientific Procedures) Act 1986 Amendment Regulations 2012, incorporating the EU Directive 2010/63/EU, which conforms to the Guide for the Care and Use of Laboratory Animals published by the US National Institutes of Health (NIH publication No. 85-23, revised 1996). Approval for this work was obtained from the Animal Welfare and Ethics Review Board (AWERB) of Imperial College London.

MI was performed in rats by left anterior descending coronary artery ligation^10^. Rats were kept for 16 weeks after MI surgery to allow development of heart failure condition. RVMs were isolated using a standard enzymatic protocol as described previously^40^, full details of myocyte isolation can be found in supplementary materials.

### Sarcolemma membrane structure characterization

Surface topography of live myocytes was visualized by scanning ion conductance microscopy (SICM), which uses a glass nanopipette as a sensitive probe (ICAPPIC Ltd, London, UK)^19^. Z-groove ratio was calculated as a total Z-groove length observed on the image normalized by the predicted value^19^. TAT network was visualized in live myocytes via Di-8-ANEPPS membrane staining. Analysis of TAT network was performed as described before^17,18^. Detailed description of the analysis can be found in supplementary materials.

### Super-resolution scanning patch-clamp

After visualization of the myocyte surface topography, the scanning pipette was clipped according to the established protocol^11^. The pipette was then positioned to a desired location and lowered until a contact with the membrane was established and a high resistance seal achieved. Single LTCC recordings were performed using cell-attached patch-clamp as described in detail previously^11^. Full details of the protocols and solutions can be found in supplementary material.

### Measuring cell contraction and calcium transient

Cardiomyocytes attached to laminin were loaded with 1uM of Fura-2AM. CytoCypher system setup and operation has been previously described^41^. Cells were paced at 0.5 Hz and sarcomere shortening and calcium transient were recorded for multiple cells selected. The data was analyzed using Transient Analyses Tool Software (IonOptix LLC, Westwood, MA, USA).

### Optical mapping of calcium activity

Ca^2+^ optical mapping in cells loaded with the Ca^2+^-sensitive fluorescent dye Fluo-4AM was performed via CMOS camera ULTIMA-L (SciMedia, , Costa Mesa, CA, USA) at 500 fps, 1.5–2 μm/pixel, connected to an inverted Nikon Eclipse Ti microscope^42^. All spontaneous Ca^2+^ releases were divided into local (occupying a portion of the cell surface and attributed to discrete clusters of Ca^2+^ sparks) and propagated waves, that propagate though the whole surface. Single Ca^2+^ sparks were monitored using line-mode of the confocal microscope^10^. The analysis of Ca^2+^ sparks images was performed using SparkMaster plugin in FIJI with a determination criterion of 4.2. Full details of can be found in supplementary material.

### Statistical analysis

All graphs were produced and statistical analysis was performed using GraphPad Prism 6 (GraphPad Software, USA). Normality was tested using the Kolmogorov–Smirnov test. Statistical differences were assessed with Student t-test, Mann–Whitney test, one-way analysis of variance (ANOVA) and Kruskal–Wallis test. All data are expressed as mean ± standard error of mean (S.E.M.). A value of P < 0.05 was considered statistically significant.

## Supplementary Information

Supplementary Information.

## Data Availability

The datasets generated during and/or analyzed during the current study are available from the corresponding author on reasonable request.
