# Supplementary material for: Local hyperactivation of L-type Ca2+ channels increases spontaneous Ca2+ release activity and cellular hypertrophy in right ventricular myocytes from heart failure rats
Source: Sci Rep. 2021 Mar 1;11:4840. doi: 10.1038/s41598-021-84275-w (PMC7921450; doi:10.1038/s41598-021-84275-w)
Supplement: Supplementary file 1 — Supplementary Information. [file 41598_2021_84275_MOESM1_ESM.pdf]

Supplementary Information

Title: Local hyperactivation of L-type Ca^{2+} channels increases spontaneous Ca^{2+} release activity and cellular hypertrophy in right ventricular myocytes from heart failure rats.

Authors' names: Roman Y. Medvedev, PhD[1,2,3] , Jose L. Sanchez-Alonso, PhD[1], Catherine A Mansfield, PhD[1], Aleksandra Judina, MS[1], Alice J. Francis, MS[1], Christina Pagiatakis, PhD[4], Natalia Trayanova PhD[5], Alexey V. Glukhov, PhD[3], Michele Miragoli, PhD[4,6], Giuseppe Faggian, MD[2], Julia Gorelik, PhD[1*]

From:
[1] National Heart and Lung Institute, Imperial College London, Du Cane Road London W12 0NN, United Kingdom;
[2] Dipartimento di Cardiochirurgia, Università degli Studi di Verona, Ospedale Borgo Trento, P.le Stefani 1, 37126 Verona, Italy;
[3] Department of Medicine, Cardiovascular Medicine, University of Wisconsin-Madison School of Medicine and Public Health, Madison, Wisconsin 53705, USA;
[4] Humanitas Clinical and Research Center - IRCCS, Rozzano (MI), Italy;
[5] Department of Biomedical Engineering and Alliance for Cardiovascular Diagnostic and Treatment innovation, Johns Hopkins University, Baltimore, USA;
[6] Dipartimento di Medicina e Chirurgia, Università degli Studi di Parma, Via Gramsci 14, 43124 Parma, Italy.
* j.gorelik@imperial.ac.uk

Supplementary Methods

Animals and ethics work

All animal procedures were performed in accordance with the criteria of Animals in Scientific Procedures Act 1986 (ASPA, 1986). The male outbred, Sprague Dawley rats (rattus norvegicus) were obtained from Charles River (Harlow, UK). Investigations of the remodelling of right ventricular cardiomyocytes during heart failure were performed on a rat model of chronic myocardial infarction (MI). 16 control and 14 MI rats from 6 to 8 month old were used. The model was produced as previously described[1] via ligation of the left anterior descending coronary artery. Briefly, rats were anaesthetized with isoflurane and placed in ventral recumbence. The thorax was shaved and sterilized. Then a left thoracotomy was performed, and the left anterior descending coronary artery was ligated. The chest was sewn up and the animal was placed back for recovery. After 16 weeks the hearts were explanted, weighed, and prepared for cell isolation.

Isolation of myocytes

RV cardiomyocytes were isolated according to the protocol of enzymatic isolation adopted from [2,3]. Briefly, the heart was cannulated in a Langendorff perfusion system for 5 min with Krebs-Henseleit (KH) buffer (containing in mM: 119 NaCl, 4.7 KCl, 0.94 $MgSO_4$, 1 $CaCl_2$, 1.2 KH_2PO_4, 25 $NaHCO_3$, 11.5 glucose; 95% O_2, 5% CO_2). The solution was changed to low calcium buffer (containing in mM 120 NaCl, 5.4 KCl, 5 $MgSO_4$, 0.15 $CaCl_2$, 5 pyruvate, 20 glucose, 20 taurine, 10 HEPES, 5 nitrilotriacetic acid; 100% O_2) for 5 min to stop the beating of the heart. Next, the heart was perfused with the same low calcium buffer but containing 1mg/ml Collagenase II and 0.6mg/ml Hyaluronidase for 10 min. After that, the heart was removed from the perfusion system, and RV was chopped out and placed in enzymatic solution composed of mM (0.15 $CaCl_2$, 120 NaCl, 5.4 KCl, 5 $MgSO_4$, 5 pyruvate, 20 glucose, 20 taurine, 10 HEPES, 5 nitrilotriacetic acid; 100% O_2) with Collagenase and Hyluronidase for 30 min under constant agitation. When the digestion was finished, cell suspension was filtered and centrifuged at 1000 rpm. Cell pellet was re-suspended in the fresh enzyme-free buffer.

Transverse-axial tubule (TAT) system labelling with Di-8-ANNEPS.

Freshly isolated cardiomyocytes were plated on MatTek dishes coated with laminin (Sigma-Aldrich). The TAT network was stained by adding 10 µM Di-8ANEPPS in a Hanks Balanced Salt Solution (without Ca^{2+} and Mg^{2+}) for 2 min as described elsewhere[1,4]. After washout of the dye, Z stack images of the cardiomyocytes internal TAT network were obtained with a Zeiss LSM 710 confocal microscope. Analysis of TAT density and power of regularity was performed in Fiji as described previously in [1]. Algorithm for TAT density and regularity analysis is shown in Supplementary Fig. 1. It includes selection of squares from several z-planes of the stack. Then, the images were binarized by automatic thresholding algorithm of Fiji to calculate the density of black pixels as TAT system density. The transversal tubules regularity was determined using a custom Matlab (The MathWorks) code which counts the Fast-Fourier transform of the waveform of the image(steps 7-9 from Supplementary Fig. S1)[1,4].

The directional analysis of TAT network was performed according to the protocol of Wagner et al[5]. The main steps of this protocol are presented in Supplementary Fig. S2. Briefly, the binarized TAT picture was produced as described previously. It was skeletonized by Fiji built-in plugin (Skeletonize 2D/3D) and the length of the skeleton per area was counted. After the skeletonization, the directionality analysis was performed in Fiji using the “Directionality” built-in plugin. It produced the histogram distribution of tubules along directions. The mean fraction of tubules

aligned in axial (0 ± 10°) and transverse (90 ± 10°) directions were obtained by the sum of the histograms of tubules aligned in axial and transverse directions.

Measuring cell contraction and calcium transient with CytoCypher

Cardiomyocytes attached to laminin were loaded with 1µM of Fura-2AM in Hank's balanced salt solution (HBSS) with 1mM Ca^{2+} and 1mM Mg^{2+} in the dark for 45 min at 37°C. The cells were then washed and kept in HBSS with 1mM Ca^{2+} and 1mM Mg^{2+} for additional 20 min in the dark at 37°C. The cells were then washed with fresh HBSS with 1mM Ca^{2+} and 1mM Mg^{2+} and used on the CytoCypher system (IonOptix LLC, Westwood, MA, USA). CytoCypher system setup and operation used for this study was previously described [6]. Dish with the cells was placed over the objective and electrodes were put into the solution. Cells were paced at 0.5Hz for 10 minutes to allow them to normalize. Cell sarcomere shortening and Ca^{2+} transient were recorded for each cell selected. The data was analyzed using Transient Analysis Tool Software (IonOptix LLC, Westwood, MA, USA). As described previously three main parameters were analysed: percentage of sarcomere shortening, time to 90% peak (TTP90) and time to 90% baseline (TTB90).[6]

Scanning Ion Conductance Microscopy (SICM)

SICM is a non-contact microscopy that scans the topography of the surface using an ion current through the nanopipette with a feedback loop, as described previously[4]. Briefly, freshly isolated cells were plated on the laminin-coated plastic dishes in the external recording solution (containinig in mM: 120 K- gluconate, 25 KCl, 2 $MgCl_2$, 1 $CaCl_2$, 2 EGTA, 10 Glucose, 10 HEPES, pH=7.4 was adjusted with NaOH). Cells were scanned by 80-100nm diameter nanopipettes at room temperature. The analysis of membrane surface Z-groove organization was made by calculating the z-groove ratio as described previously[7]. This parameter was determined as a ratio between the total lengths of z-grooves observed on the single 100 square micron image to the estimated value from the image with an ideal z-groove structure.

Super Resolution Patch Clamp technique

This methodology presents a combination of SICM and conventional cell-attached patch-clamp. It has been used previously to study microdomains of different cells [3,8]. Briefly, SICM was used for the visualisation of the surface topography of lived myocytes. The pipette was moved out of the cell surface and its tip was precisely clipped (up to 20-40 MΩ) to increase the area of patching, as described in [3,8]. Then pipette was positioned back to the desired position on the cell surface and lowered down until the contact with the membrane has been established. The cells were bathed in solution contained in mM: 120 K- gluconate, 25 KCl, 2 $MgCl_2$, 1 $CaCl_2$, 2 EGTA, 10 Glucose, 10 HEPES, pH=7.4 was adjusted with NaOH. The pipette solution included in mM: 90 $BaCl_2$, 10 sucrose, 10 HEPES, pH=7.4 adjusted with tetraethylammonium hydroxide (TEA-OH). The channel activity was recorded by at least 100 sweeps from holding potential -96.7 mV to -6.7 mV. Liquid junction potential, calculated to be -16.7 mV, was corrected from the data shown. The open probability (P_o) of the channel was calculated using Clampfit 10.3 function by averaging 20 sweeps at -6.7 mV for each channel. All-points histograms were constructed from single-channel opening amplitudes recorded at -6.7mV, detected by pClamp software single channel search tool. Data was fitted to a multicomponent Gaussian curve.

The occurrence of the LTCC at T-tubule and crest was calculated as the ratio of the number of patches contained LTCC to the total number of successful patches in selected myocyte area. For the conductance measurements of the single LTCC the channel activity was recorded at potential range from -26.7mV to 13.3mV with a 10mV step from a holding potential of -96.7mV. The conductance was calculated as a slope of the linear regression of the voltage-current plot.
The effect of PKA blockade on the LTCC properties was measured in MI RVMs pre-incubated for 5 min with 10uM of H-89 in external solution. Recordings on one dish were performed for less than 30 min to exclude LTCC degradation. Similar protocols were applied to measure LTCC Po, amplitude and conductance.

Spontaneous Ca^{2+} waves measured in single myocytes

Spontaneous Ca^{2+} waves and induced Ca^{2+} transients were recorded by optical mapping as described previously[9,10]. Cardiomyocytes were loaded with 10 µmol/L Ca^{2+}–sensitive fluorescent dye fluo4-AM for 30 min. During experiments the cells were superfused with a HBSS (with 1mM Ca^{2+} and 1mM Mg^{2+}) at 37°C. Fluo-4AM was excited with 488 nm LED lamp and the fluorescence was collected through a 520 nm filter by MiCAM Ultima-L CMOS camera (SciMedia, USA Ltd, Costa Mesa, CA) at frequency 500 fps.
Spontaneous Ca^{2+} waves were studied in single cells after 1 min of 4 Hz field stimulation to maintain the same sarcoplasmic reticulum loading. Wave frequency was counted by the number of spontaneous Ca^{2+} events occurred during the 8-16 sec period after the termination of the pacing. Waves were assigned as propagated when it covered more than 90 % of the cell surface; otherwise, the wave was assigned as local. To elucidate the effect of PKA blockade on the frequency of spontaneous Ca^{2+} waves, the dish with the cells were firstly imaged for the baseline Ca^{2+} waves frequency and then the perfusion buffer was changed to HBSS contained 10uM of H-89. The recordings were performed after 5 min of perfusion with H-89.

Ca^{2+} spark measurements

Spontaneous Ca^{2+} sparks were analysed in single RV myocytes loaded with Fluo-4AM as described above and in [1]. Briefly, after 30 min of incubation with the dye, cardiomyocytes were resuspended with normal Tyrode solution containing 1mM of $CaCl_2$. During experiments cells were continuously superfused with normal Tyrode solution at 37°C. Fluo-4AM was excited by the 488nm wavelength of the argon laser and emission was collected through the 520nm filter. Ca^{2+} sparks activity was recorded in the line mode of a confocal microscope with a line places along the main axes of the cell (0.2ms time resolution and 0.2-0.4µm spatial resolution). The role of PKA blockade in modulation of Ca^{2+} spark properties was analysed by measuring Ca^{2+} activity frequency before and after 5 min of incubation with 10uM of H-89.
The analysis of Ca^{2+} sparks was made with the SparkMaster plugin[11] of ImageJ with the detection criteria set 4.2. The mass of Ca^{2+} sparks was calculated accordingly to Kolstad et al[12] by multiplication of spark amplitude by full width half maximum (FWHM) and full duration half maximum (FDHM).

Supplemental References

1. Lyon, A. R. *et al.* Loss of T-tubules and other changes to surface topography in ventricular myocytes from failing human and rat heart. *Proc. Natl. Acad. Sci.* **106**, 6854–6859 10.1073/pnas.0809777106 (2009).
2. Sato, M., O'Gara, P., Harding, S. E. & Fuller, S. J. Enhancement of adenoviral gene transfer to adult rat cardiomyocytes in vivo by immobilization and ultrasound treatment of the heart. *Gene Ther.* **12**, 936–941 10.1038/sj.gt.3302476 (2005).
3. Sanchez-Alonso, J. L. *et al.* Microdomain-Specific Modulation of L-Type Calcium Channels Leads to Triggered Ventricular Arrhythmia in Heart Failure. *Circ. Res.* **119**, 944–945 10.1161/CIRCRESAHA.116.308698 (2016).
4. Schobesberger, S. *et al.* T-tubule remodelling disturbs localized β2-adrenergic signalling in rat ventricular myocytes during the progression of heart failure. *Cardiovasc. Res.* **113**, 770–782 10.1093/cvr/cvx074 (2017).
5. Wagner, E. *et al.* Stimulated Emission Depletion Live-Cell Super-Resolution Imaging Shows Proliferative Remodeling of T-Tubule Membrane Structures After Myocardial Infarction. *New Methods Cardiovasc. Biol. Stimul.* **111**, 402–14 10.1161/CIRCRESAHA.112 (2012).
6. Wright, P. T., Tsui, S. F., Francis, A. J., MacLeod, K. T. & Marston, S. B. Approaches to High-Throughput Analysis of Cardiomyocyte Contractility. *Front. Physiol.* **11**, 612 10.3389/fphys.2020.00612 (2020).
7. Gorelik, J. *et al.* A novel Z-groove index characterizing myocardial surface structure. *Cardiovasc. Res.* **72**, 422–429 10.1016/j.cardiores.2006.09.009 (2006).
8. Bhargava, A. *et al.* Super-resolution scanning patch clamp reveals clustering of functional ion channels in adult ventricular myocyte. *Circ. Res.* **112**, 1112–1120 10.1161/CIRCRESAHA.111.300445 (2013).
9. Glukhov, A. V. *et al.* Direct evidence for microdomain-specific localization and remodeling of functional L-type calcium channels in rat and human atrial myocytes. *Circulation* **132**, 2372–2384 10.1161/CIRCULATIONAHA.115.018131 (2015).
10. Miragoli, M. *et al.* Microtubule-Dependent Mitochondria Alignment Regulates Calcium Release in Response to Nanomechanical Stimulus in Heart Myocytes. *Cell Rep.* **14**, 140–151 10.1016/j.celrep.2015.12.014 (2016).
11. Picht, E., Zima, A. V., Blatter, L. A. & Bers, D. M. SparkMaster: automated calcium spark analysis with ImageJ. *AJP Cell Physiol.* **293**, C1073–C1081 10.1152/ajpcell.00586.2006 (2007).
12. Kolstad, T. R. *et al.* Ryanodine receptor dispersion disrupts Ca 2+ release in failing cardiac myocytes. *Elife* **7**, 1–24 10.7554/eLife.39427 (2018).

Supplementary figures

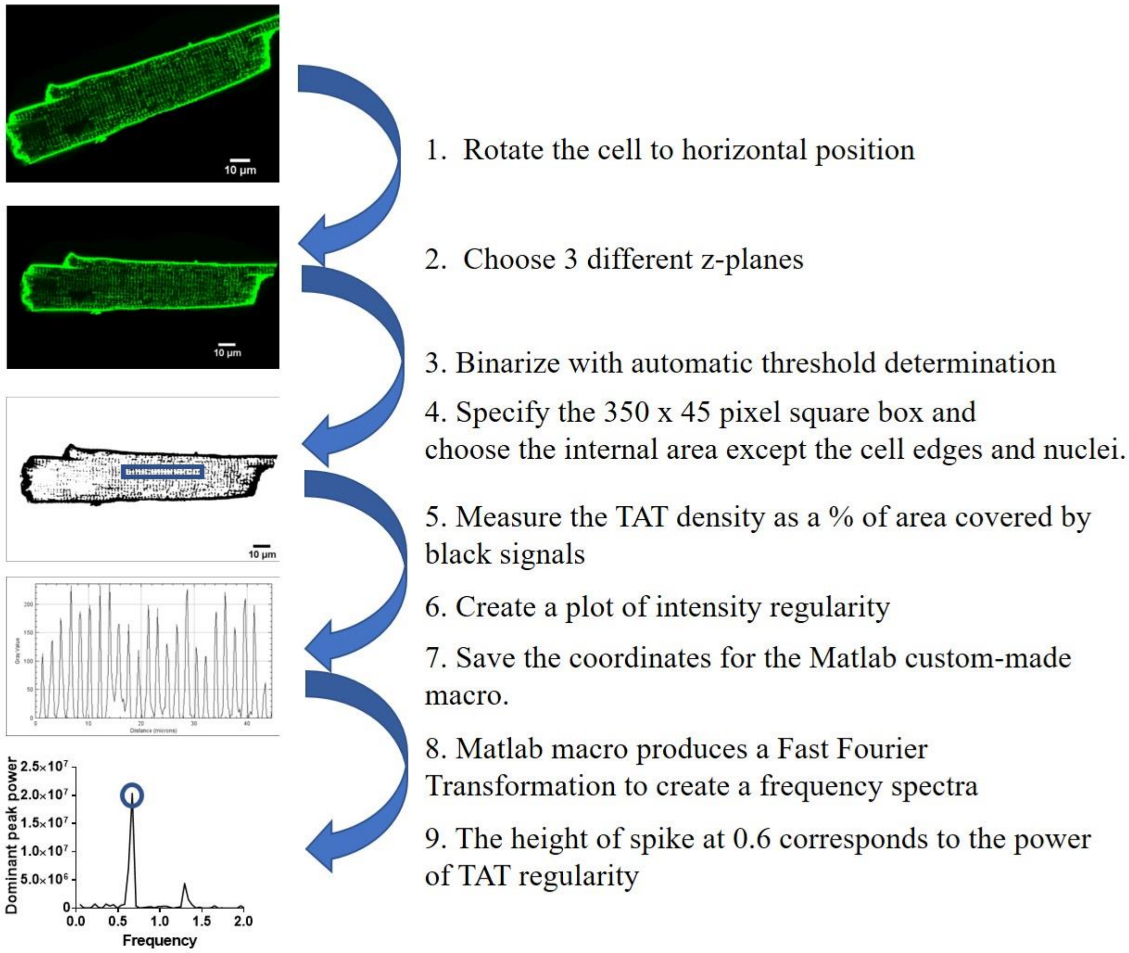

Supplementary figure 1. Image processing algorithm for TAT network density and regularity analysis performed Fiji and Matlab. Scale bar- 10µm.

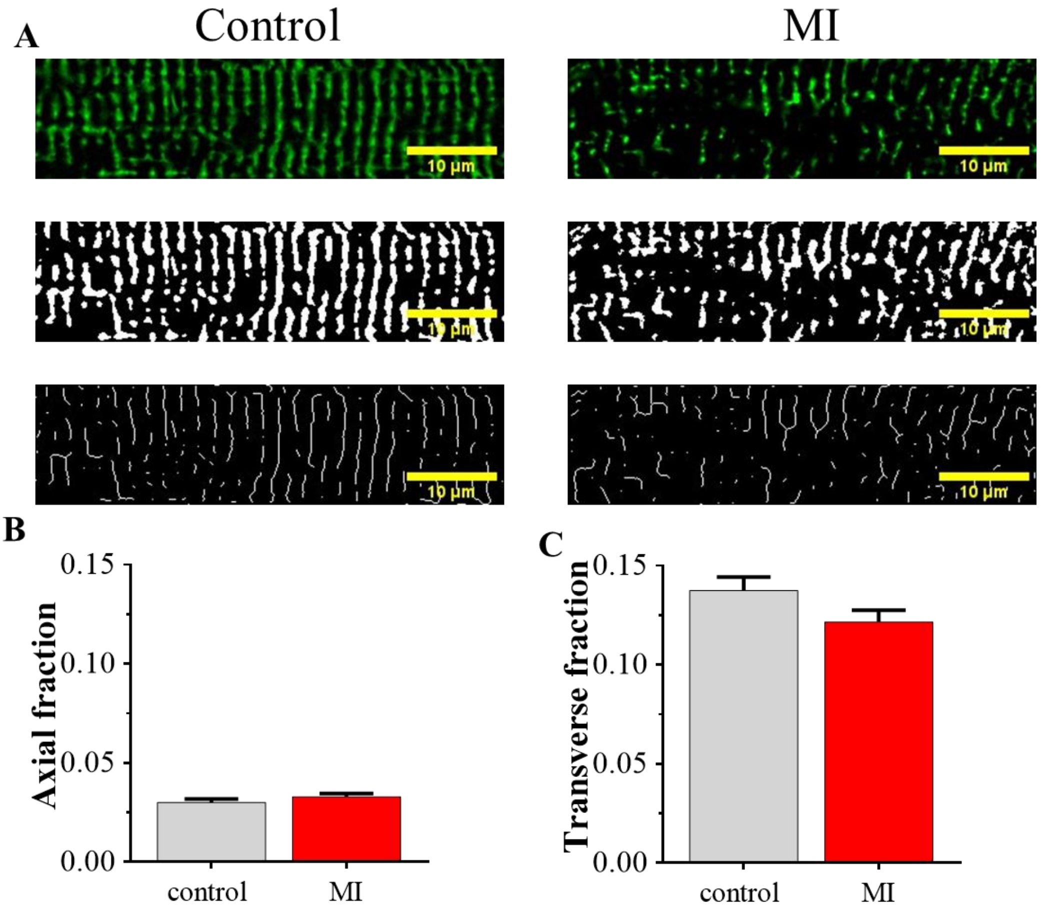

Supplementary figure 2. TAT network directionality analysis steps. A) 50x15 microns cuts from control and MI RVM Di-8ANEPPS images unproceded (top), binarized (middle) and skeletonized (bottom). **B)** Statistical analysis of frequency of axial and **C)** transverse tubules at TAT network of RVM isolated from control and MI rats (Control n=40 cells from 5 rats, MI n=62 cells from 6 rats).

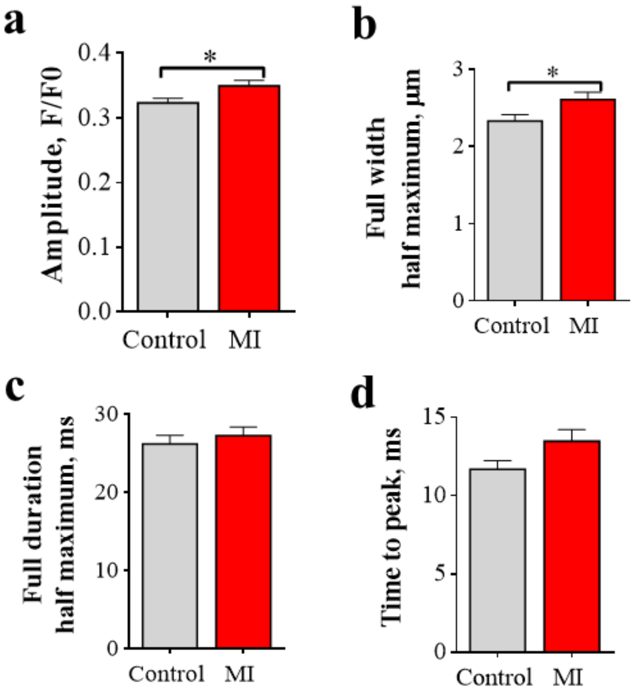

Supplementary figure 3. Statistical analysis of average Ca^{2+} spark properties: **(a)** amplitude, **(b)** full width half maximum, **(c)** full duration half maximum and **(d)** time to peak measured in control and MI RVM. (Control n=26 cells from 4 rats, MI n=21 from 4 rats, *P<0.05, by Mann-Whitney test).

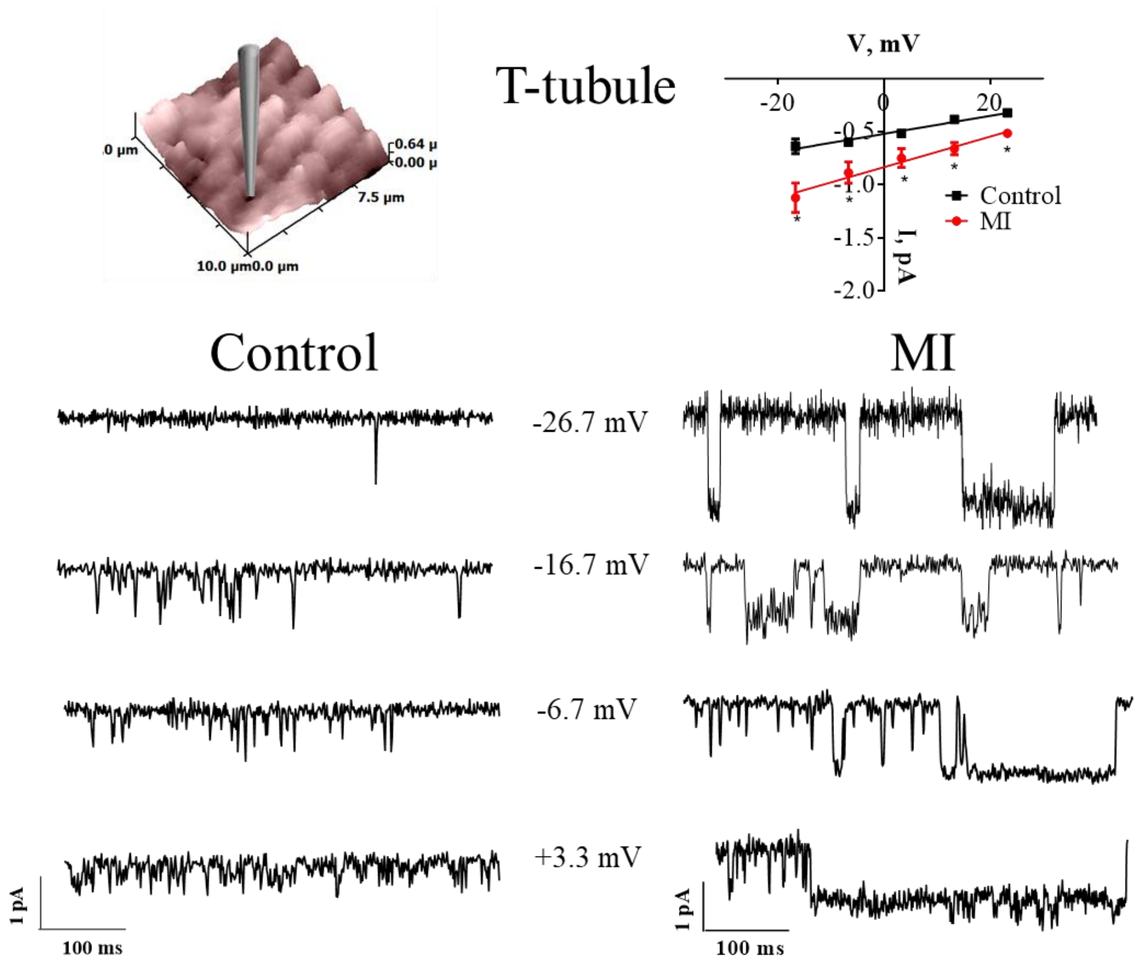

Supplementary figure 4. Representative traces of LTCC recorded in T-tubule of control and MI RVMs and corresponding current-voltage curves. Recordings were performed from holding potential of -96.7 to a range of potentials from -26.7 to 3.3 mV. Control T-tubule n=7; MI T-tubule n=10, *P<0.05, by Student t-test.

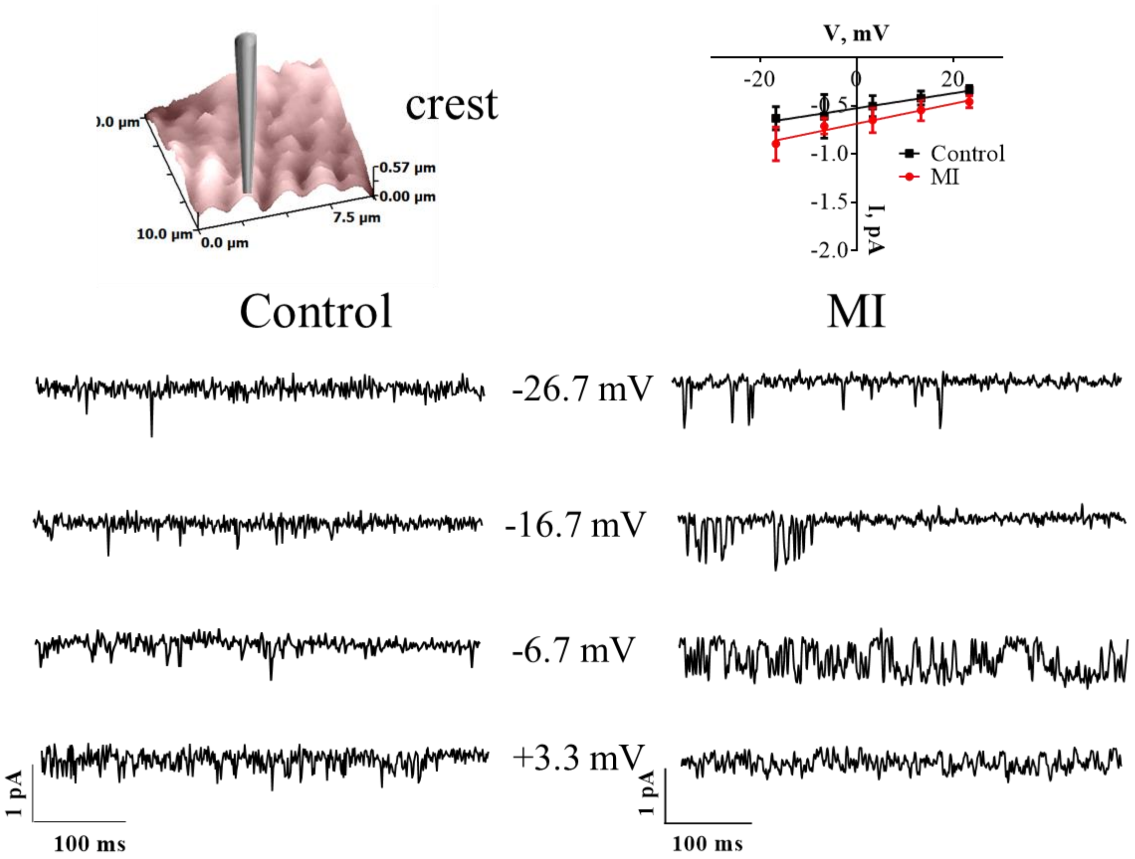

Supplementary figure 5. Representative traces of LTCC recorded in crest of control and MI RVMs and corresponding current-voltage curves. Recordings were performed from holding potential of -96.7 to a range of potentials from -26.7 to 3.3 mV. Control crest n=10; MI crest n=12.

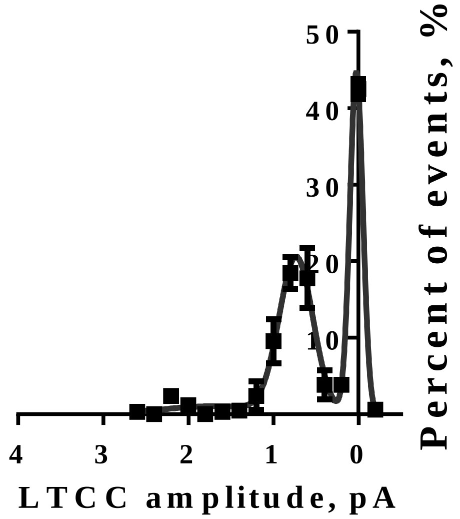

Supplementary figure 6. Amplitude histogram of LTCC (dots) recorded in MI RVMs treated with H-89 at depolarizing step of -6.7mV and fitted with multicomponent Gaussian functions (connecting curves). Histograms were constructed from 5 cells.
